# Ultrasonic-aided fabrication of gold nanofluids

**DOI:** 10.1186/1556-276X-6-198

**Published:** 2011-03-07

**Authors:** Hui-Jiuan Chen, Dongsheng Wen

**Affiliations:** 1 School of Engineering and Materials Science, Queen Mary University of London, London, UK

## Abstract

A novel ultrasonic-aided one-step method for the fabrication of gold nanofluids is proposed in this study. Both spherical- and plate-shaped gold nanoparticles (GNPs) in the size range of 10-300 nm are synthesized. Subsequent purification produces well-controlled nanofluids with known solid and liquid contents. The morphology and properties of the nanoparticle and nanofluids are characterized by transmission electron microscopy, scanning electron microscope, energy dispersive X-ray spectroscope, X-ray diffraction spectroscopy, and dynamic light scattering, as well as effective thermal conductivities. The ultrasonication technique is found to be a very powerful tool in engineering the size and shape of GNPs. Subsequent property measurement shows that both particle size and particle shape play significant roles in determining the effective thermal conductivity. A large increase in effective thermal conductivity can be achieved (approximately 65%) for gold nanofluids using plate-shaped particles under low particle concentrations (i.e.764 μM/L).

## Introduction

In recent years, there have been intensive efforts in the synthesis and application of nanomaterials in different fields, from energy to biomedicine sectors. Widespread interest has been generated in tailoring gold nanoparticles (GNP) for non-invasive medical applications, either as a heating or a targeting agent for detection, diagnosis, or treatment [1]. GNPs are popularly chosen because of their unique physical and chemical properties, such as high conductivity (for both heat and electricity), easy functionalization and bio-compatibility, as well as prior clinical experience of gold-based pharmaceuticals. One example is in cancer therapy where functionalized GNPs are proposed as potential agents for non-invasive thermal treatment, and the feasibility has been proven in preliminary studies on non-targeted particles *in vitro*, and later *in vivo *[2,3]. Almost all these applications involve delivering bio-modified nanoparticles to malignant cells and rapidly heating nanoparticles with an external source such as laser, ultrasound, or an electromagnetic wave to produce a therapeutic effect or to release drugs [3]. The interaction of nanoparticles with the external source and subsequent heating effect are fundamental for the successful deployment of these novel techniques, where the thermophysical properties of nanoparticle suspensions play a key role.

The last decade witnessed a quick development of nanofluids field especially on its application in heat transfer field. While the original idea of nanofluids was to enhance the thermal conductivities of some typical heat transfer fluids including water, mineral oil, and ethylene glycol, the influence of nanoparticles has been found to be more profound than the mean thermal conductivity effect for application in different situations. This field has developed very rapidly in the past few years. However, a large number of controversies have been reported, ranging from basic properties such as thermal conductivity, viscosity, and single phase convection to boiling heat transfer [4]. It has been suggested that current uncertainties on the content of nanofluids, including both solid and liquid phases, are one of the main reasons responsible for many of the observed controversies and inconsistencies [4,5], highlighting the importance of nanoparticle synthesis and nanofluids formation.

Two methods are generally used for nanofluid formulation, namely, the top-down method through size reduction (the two-step method), and the bottom-up approach through simultaneous production and dispersion of nanoparticles (the one-step method) [5,6]. For the two-step method, nanoparticles are either synthesized or purchased first in the form of dry powders, and the nanofluid formulation process involves properly separating the aggregated dried particles into individual particles and keeping them from re-agglomeration under suitable ionic or surfactant conditions. How well the final dispersion is achieved depends on (i) the degree of agglomeration of the dried nanoparticles (which depends on the nature of the manufacturing, handling and storage process), (ii) the shear forces applied in separating agglomerations (weakly bonded agglomerates could be broken to their primary sizes by high shearing, but strongly bonded ones are difficult to be separated), and (iii) the liquid environment (pH or ionic conditions to keep separated particles from re-aggregation). As a result of these complicated factors and a lack of detailed characterization of the content and morphology of the liquid and solid phases, it is very difficult to compare the result from one study with another even using same nanoparticles. The formulation of nanofluids by the bottom-up approach through physical or chemical reactions has been gaining increasing interests [7]. Such a method has been practiced for a long time in the colloid industry, i.e., colloidal gold. A number of other nanofluids have also been formulated including copper and iron nanofluids through a modified physical vapor deposition method and a hydrothermal chemical reduction of salts [8,9]. For nanofluids formulated through the one-step method, the stability of nanofluids can be improved through proper surface functionalization without involving mechanical facilities. This approach, however, suffers the problem of impurities, i.e., residual reactants are generally left in the nanofluids because of incomplete reaction or stabilization. It is difficult to elucidate the nanoparticle effect without eliminating this impurity effect.

Using gold nanofluids as an example, this study aims to engineer a number of nanofluids with different particle sizes and shapes (spherical and plate shaped) under controlled liquid conditions, and characterize their thermophysical properties accordingly. Gold nanoparticles can be generally synthesized by the citrate reduction (CR) method [10], the Brust-Schiffrin method [11], and the modified Brust-Schiffrin method that contains different sulfur-containing ligands. The size of GNPs can be tuned by controlling the ratio of thiol or other ligands to Au ions used in the synthesis. In the nanofluids community, GNPs have been investigated only by a few studies because of its high cost [12]. To achieve better control of nanoparticle morphology, sonochemical technique, especially ultrasound will be used in this study for particle shape control. Such a technique is based on the acoustic cavitation: the rapid collapse of small gas bubbles in sonicated solutions promotes the reaction to be more homogeneous. A number of metallic-based spherical particles with narrow size distribution, such as Au, Ti, Pt, Pd, Fe, MnO_2_, and CdS, have been successfully produced ultrasonically [13-15]. The experiment of using sonication technique to improve the quality of other-shaped nanomaterials, however, is seldom reported, which will be another novel point of this study.

## Gold nanofluids formulation

Four groups (Group A, B, C and D below) of gold nanomaterials, with particle size ranging from 10 to 300 nm in spherical and plate shapes, will be synthesized using different methods.

### Gold nanofluids with spherical particles

#### CR method as the control group (Group A)

The modified CR method [16] was first used to produce spherical GNPs as the control group. In this method, 5.0 × 10^-6 ^mol of HAuCl_4 _in 190 ml of DI water was heated until boiling. While the solution was kept heated and stirred by a magnetic blender, 10 ml of 0.5% sodium citrate was added. The solution was kept stirring for the next 30 min until the reaction was completed.

#### CR with ultrasonic irradiation method (Group B)

Same chemicals as the conventional CR were used in this method. 5.0 × 10^-6 ^mol of HAuCl_4 _in 190 ml of DI water was heated until boiling. It was then subjected to heating, and stirred using a magnetic blender; 10 ml of 0.5% sodium citrate was added into the solution until its color changed to wine-red. To examine the influence of controlling factors, the solution with wine-red color was further divided into four groups at different temperature and ultrasonic or stirring time:

▪ The first solution was placed in the ultrasonic bath at 80°C for 30 min;

▪ The second solution was placed in the ultrasonic bath at 80°C for 45 min;

▪ The third solution was stirred and heated at 100°C by magnetic blender for 10 min and was then placed in the ultrasonic bath at 80°C for 20 min;

▪ The fourth solution was stirred and heated at 100°C by magnetic blender for 20 min and was then placed in the ultrasonic bath at 80°C for 10 min.

### Gold nanofluids with plate-shaped particles

#### Synthesis of gold nanoplates through CR at room temperature (Group C)

Similar method as proposed by Huang et al. [17] was used for synthesizing gold nanoplates. Based on the CR method, 1.3 ml of 1% HAuCl_4 _was added to 100 ml of DI water at 25°C, and stirred by a magnetic blender for 1 min. 0.4 ml of sodium citrate (38.8 mmol/l) was then introduced in the HAuCl_4 _solution and stirred for the next 30 min.

The resultant solution was exposed under natural light in the laboratory for 16 h.

#### Synthesis of gold nanoplates through CR at room temperature with the aid of ultrasonication (Group D)

Based on CR method, 1.3 ml of 1% HAuCl_4 _was added to 100 ml of DI water at room temperature and was sonicated for 1 min. 0.4 ml of sodium citrate (38.8 mmol/l) was then added in the HAuCl_4 _solution. The resultant solution was divided into five groups, each treated further with ultrasonication times of 10, 20, 30, 45, and 60 min. Subsequently, these resultant solutions were exposed under natural light in the laboratory for another 16 h, which changed the solution color to cloudy blue.

The synthesized gold nanoproducts are separated by the centrifugation method, re-dispersed into DI water, and further purified through membrane filters for 4-6 days where some residual reactants and stabilizer are diffused away. The purified nanofluids are stored for further morphological and property characterization.

### Gold nanofluids characterization

The primary size and shape of all gold nanomaterials are identified using a transmission electron microscopy (TEM) and a scanning electron microscope (SEM) equipped with an energy dispersive X-ray spectroscope (EDX). In this process, the TEM was performed using a Jeol JEM-2010 electron microscope at a bias voltage of 200 kV, and SEM image was taken at 10/20 kV accelerating voltage. The particle size distribution in liquid was identified by a dynamic light scattering (DLS) device (Malvern nanosizer). The crystal structure and elemental information were provided by X-ray diffraction spectroscopy. The size distribution of GNP in DI water was measured by a Zetasizer Nano-Z (Malvern Instruments Ltd, Worcestershire, UK) with a minimum of 15 runs being performed. Each result was the average of three consequent measurements. A KD2 Pro Thermal Properties Analyzer device was employed to measure the thermal properties of gold nanomaterial dispersions at different concentrations (1.1, 11.1, 33.3, 330, 764 μM/l). DI water as a control group was measured, and each sample was repeated at least three times.

## Result and discussion

### Characterization of nanofluids

#### Groups A and B: spherical particles and particle size control

**Figure 1:**
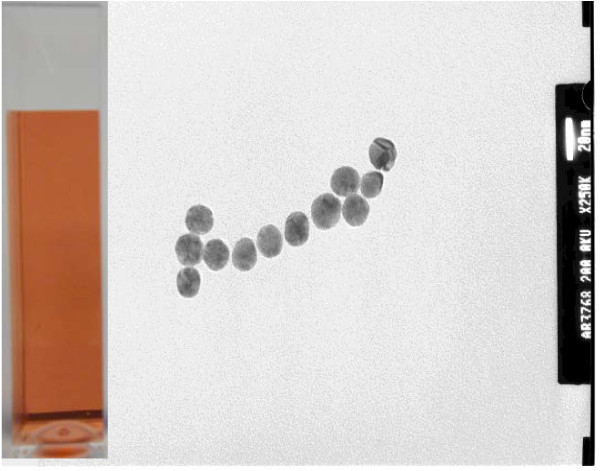
**TEM image of the control sample (sodium citrate concentration 0.5%, inset: resulting dispersion of red-wire color)**.

**Figure 2:**
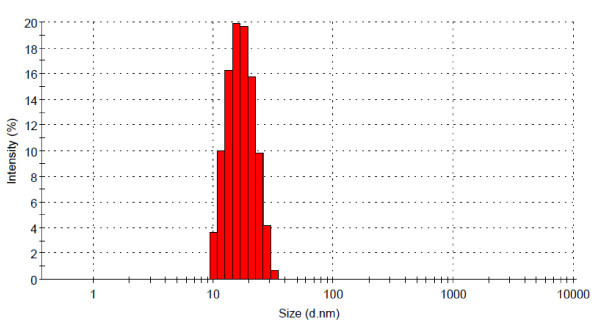
**Particle size distribution of GNPs fabricated by citrate reduction with further sonication of 45 min (measured by Malvern Zetasizer)**.

The resulting dispersion from CR method exhibits a clear wine-red color (Group A samples). TEM images of these nanoparticles are shown in Figure 1. The average size of the GNPs is approximately in the range of 15-20 nm in diameter, and the shape is spherical. The particle size distribution in the liquid phase is measured by the DLS method. A narrow size-distribution is found, typically in the range of 10-30 nm, as shown in Figure 2. It should be noted that the measured particle size in the liquid medium is generally larger than the primary particle size even under fully dispersed status (no agglomeration), as the DLS measures the hydrodynamic size of the particles, determined by the Brownian motion effect. Consequently the DLS result reveals almost a fully dispersed particle status in the liquid. Further control of the pore size of the membrane filter allows for a narrower size distribution.

Chemically, the size of GNPs can be controlled by the ratio of the reducing/stabilizing agents to the gold (III) derivatives. In this study, 15-nm GNPs were produced by adding 0.5% (wt%) of sodium citrate, which acted as a reducing agent at the beginning and a stabilizer subsequently. Larger GNPs can be engineered by using a reduced amount of sodium citrate. Stoichiometrically, 0.05% (wt%) of sodium citrate is required to reduce all the gold (III) derivatives in this sample. Incomplete reaction occurs if the sodium citrate concentration is smaller than 0.05% (wt%), and vice versa. For concentrations less than 0.5%, the amount of extra citrate ions will not be sufficient to stabilize all the GNPs, which would result in an aggregation phenomenon producing large nanoparticles. As a consequence, a general trend of GNP size reduction with the increase of sodium citrate is observed in the experiments, which is consistent with other studies. By properly controlling the ratio of sodium citrate to gold (III) derivatives, the GNP size can be engineered in the range of 10-150 nm.

**Figure 3:**
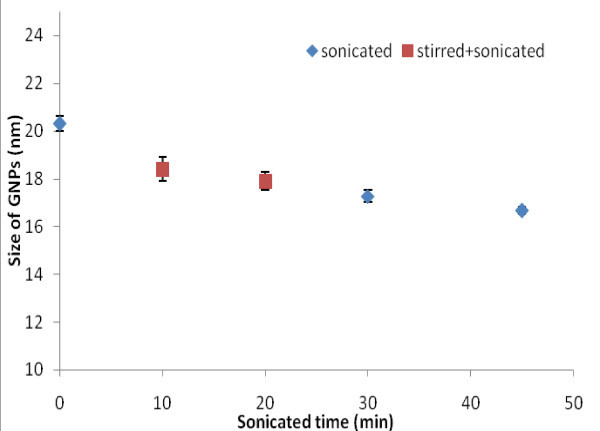
**Average size of gold nanoparticles in DI water measured by a Zetasizer (blue points show the size of GNPs sonicated for 0, 30, and 45 min, and red points present the size of GNPs using a mixed magnetic stirring and ultrasonication of total 30 min)**.

With the application of ultrasonication during the synthesis (Group B samples), the particle size becomes smaller, being reduced from approximately 20 to 16 nm as measured by Zetasizer based on the DLS method (Figure 3). The red-colored points refer to a mixed use of magnetic stirring and ultrasonication methods. The examination of the three points in the center (all having a total processing time of 30 min) shows that ultrasonication is a more powerful tool in particle size reduction as compared with the magnetic stirring. TEM images also show that the size distribution of GNPs with a mixed use of stirring and ultrasonication is not as uniform as that with pure ultrasonication under the same processing time. Increasing the ultrasonication time produces smaller particles with more regular spherical shapes, probably because of a closer to a homogeneous reaction.

#### Group C and D: gold nanoplates and size control

**Figure 4:**
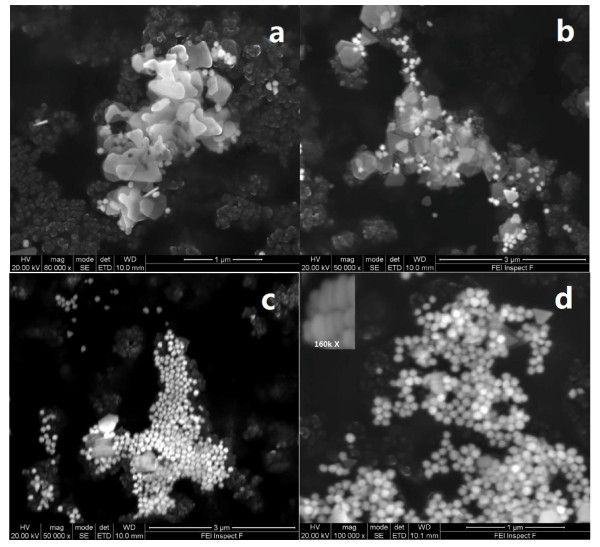
**SEM images of gold nanoplates fabricated by CR with ultrasonication of 0 min**. **(a)**, 10 min **(b)**, 20 min **(c)**, and 30 min **(d)**.

The resulting dispersion of CR produced gold nanoplates appears cloudy brown in color (Group C samples). SEM image illustrates that the main products are in plate-like shape. Figure 4a shows that these gold nanoplates are around 220-280 nm in size along their longest edge, having triangular and truncated triangular shapes with uniform edges. The particle size is not uniform, with some small gold nanoplates of about 60-70 nm appearing. The formation mechanism of CR gold nanoplates can be related to the kinetically preferred development of the redundant Au ions in the lateral direction of the small gold nuclei. The temperature has been found to have an important effect on the CR reduction rate. At 25°C, the reduction process is substantially slow and the formation changes to a kinetic-controlled mechanism that is appropriate for the production of highly anisotropic structures, which is the reason why gold nanoplates can be fabricated without additional stabilizers. Furthermore, the existence of natural light is another important feature for the formation of gold nanoplates. It is difficult to process the reaction without the exposure to natural light even if all other conditions are the same.

**Figure 5:**
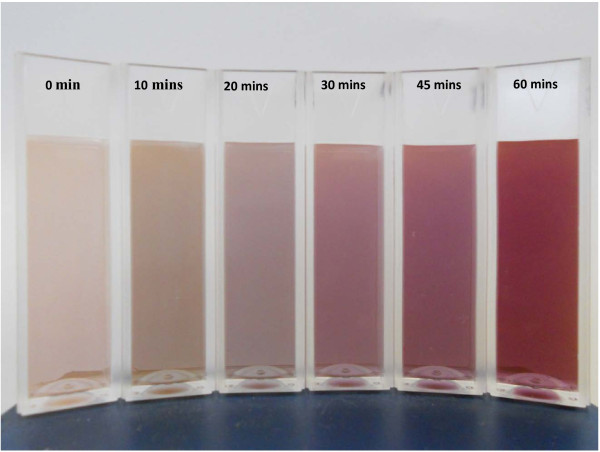
**The colors of gold nanoplate suspensions fabricated by CR with sonication time of 0, 10, 20, 30, 45, and 60 min (left to right)**.

**Figure 6:**
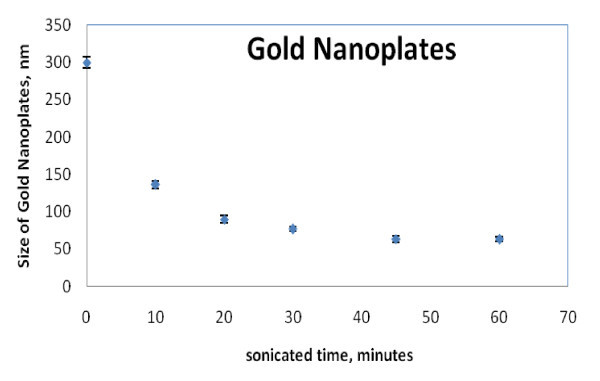
**The average size of gold nanoplates in DI water sonicated for 0, 10, 20, 30, 45, and 60 min measured by a zetasizer**.

The particle size and shape change significantly with the aid of ultrasonication (Group D samples), as shown by SEM images in Figure 4. In general, the particle becomes smaller, more regular, with more products exhibiting hexagonal shapes with the increase of ultrasonication time. Depending on the ultrasonication duration, the resultant dispersions display different colors, Figure 5. The average particle size measured by DLS method is shown in Figure 6. The particle-size reduction levels off at an approximate ultrasonication time of 45 min. Such a result demonstrates that ultrasonic irradiation is a very useful tool to engineer different particle morphologies. It can be strong enough to prompt reaction even just within 10 min, resulting in over 50% reduction in the average particle size, Figure 6. Compared with spherical particles, the application of ultrasonication to homogenize the synthesis process is more effective for gold nanoplates.

**Figure 7:**
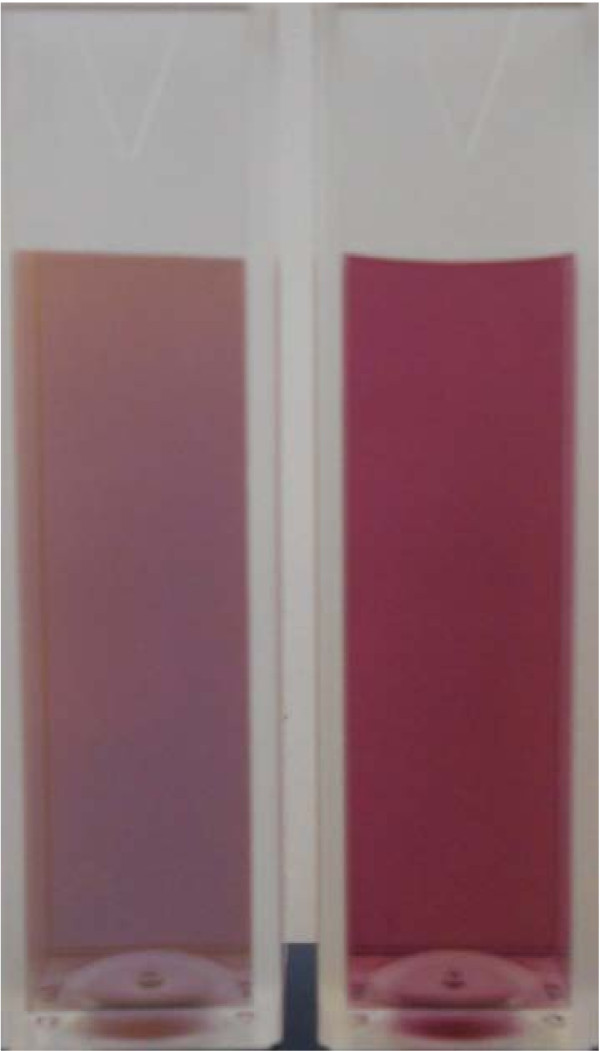
**The colors of 90-nm gold nanoplates (left) and 90-nm GNPs (right)**.

Different colors of gold nanofluids, shown in Figure 5, are due to the effect of surface plasmon resonance (SPR), an optical phenomenon arising from the collective oscillation of conduction electrons [18]. The SPR is a size-dependent phenomenon, which renders different colors for different gold nanofluids. For spherical nanoparticles, the color of gold dispersion is dark blue and purple-red for 15-nm and 90 nm particles, respectively. For gold nanoplates, Figure 5 also reveals a size-dependent color phenomenon. A comparison to spherical particles at similar size, Figure 7, shows that the dispersion color is not only dependent on particle size, but also on the particle shape. Similar results were also obtained by the Orendorff`s group [18]. Consequently, by engineering gold nanomaterials into different shapes, i.e., nanorod, nano-cage, or other anisotropic shapes, the SPR peak can be shifted from the visible light spectrum to nearly infrared regime, which can be used for many nanoparticle-mediated thermal therapies such as the plasmonic photothermal therapy (PPT) [19,20].

The effective particle size reduction by ultrasonic irradiation is because of the acoustic cavitation, which is affected by the growth and collapse of cavitation bubbles. The cavitation process introduces a disintegration of water or volatile precursors (RH) into hydrogen and hydroxyl because of the high temperature and strong pressure in collapsing cavities. Consequently the existence of an ultrasonic field enables the control of the rate of AuCl_4_^- ^reduction in an aqueous solution. The sizes of formed GNPs or gold nanoplates can be controlled by parameters, such as the temperature of the solution, the intensity, direction, and duration of the ultrasound source. Prolonged directional irradiation would cause the development of either anisotropic or aggregated Au nanoparticles.

It has been demonstrated that the synthesis of anisotropic nanostructures in the liquid phase is commonly related to two features: (1) surfactant-based soft template approach that provokes the exclusive growth direction of the nanoparticles, and (2) the selective adsorption of small molecules or polymers on specific crystal planes that controls the growth rate along a specific direction [14]. In the synthesis of gold nanoplates, ultrasonic irradiation is employed to replace magnetic blender as it can induce strong pressure in collapsing cavities locally and immediately in solution, promoting a quasi-balance growth of gold nanomaterials. The formation of gold nanoplates may be associated with the cavitation efficiency, i.e., the amount and division of bubbles, the size and lifetime of bubbles, the dynamics and shape of the collapsing bubble, as well as the resultant temperature and pressure within the cavitation bubbles. These factors would affect the final morphology of gold nanoplates, as also shown by Okitsu et al. [21].

#### Effective thermal conductivities of nanofluids

**Figure 8:**
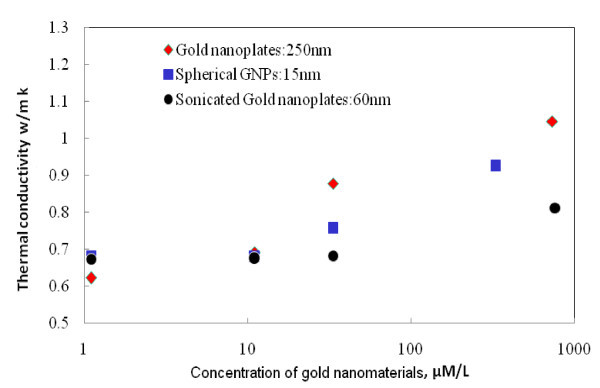
**The thermal conductivities of the gold nanomaterials (250, 60, and 15 nm)**.

Figure 8 shows the effective thermal conductivity of gold nanofluids in different concentrations of 1.1, 11.1, 33.31, 330, and 764 μM/L, respectively. The uncertainty of the thermal conductivity measurement is calibrated before use, which has an uncertainty of 8.4% within the experimental range. With the increase of particle concentrations, the effective thermal conductivities of gold nanofluids increase, exhibiting a non-linear trend, i.e., the increase is small at low concentrations but becomes significantly at over 33.31 μM/l. Figure 8 also shows that the effective thermal conductivity, *k*_eff_, is significantly affected by particle size. As the specific surface area increases with the decrease of particle size, it is expected that *k*_eff _would be higher at low particle dimensions. This is true when we compare the gold nanofluids containing 10-nm spherical nanoparticles with that of 60-nm gold nanoplates. For instance, at a concentration of 33.3 μM/L, *k*_eff _is 30% higher than the base fluid for 10-nm spherical nanoparticles whereas a 17% enhancement is observed for 60-nm gold nanoplates. In a similar study using chemical synthesized GNPs, Paul et al. [22] obtained 48% increase in the effective thermal conductivity for 0.00026 vol.% concentration with an average particle size of 21 nm. Such a trend should be maintained until the thermal conductivity of the solid particle becomes significantly size-dependent. It is well-known from physics that the thermal conductivity of a solid particle becomes smaller at lower dimensions because of the confinement of the phonon dynamics by the interface. Consequently, further increase in the specific surface area is penalized by a decrease in the particle thermal conductivities. Qualitatively, there would have an optimum particle size where a maximum increase in the effective thermal conductivity is reached. The exact optimum size is difficult to quantify as it depends on an accurate prediction of size-dependent thermal conductivity, which alone is still an active research topic, as well as the interfacial resistance between the particle and suspending liquid that will be discussed below.

In contrast to the particle size effect, when we compare the results of the 250-nm gold plates with that of spherical particle nanofluids, a reverse trend is obtained. The thermal conductivities of nanofluids containing 250-nm gold nanoplate is always higher than that of 15-nm spherical particles. The particle at the concentration of 764 μM/L reaches approximately 1.0 and 0.8 W/mK, respectively, for 250-nm gold plates and 15-nm spherical particles. Such a result shows that apart from the particle size, particle shape also plays a significant role in determining the effective thermal conductivity. While the effect of shape is small at low particle concentrations, it signifies its influences as the concentration increases. Qualitatively, such a result is consistent with a few other studies. Analytically, the Hamilton-Crosser equation [23] predicts the effective thermal conductivity of a heterogeneous mixtures by incorporating a shape factor, i.e., the higher the shape factor, the higher the predicted thermal conductivity. Experimentally, for instance, Kim and Peterson [24] also showed that different morphologies of carbon nanotubes affected effective thermal conductivity significantly, and a 37% increase in multiwalled carbon nanotube dispersion could be predicted by the Hamilton-Crosser equation with a massive shape factor of 36. Recently, the International Nanofluid Properties Benchmark Exercise (INPBE) showed that the effective thermal conductivities of alumina nanorod nanofluids (80 nm in length and 10 nm in diameter) were 45% and 30% higher than that of 10-nm spherical alumina nanofluids at concentrations of 3% and 1% volume fraction of nanomaterials, respectively [25]. A few other studies have also reached similar conclusion [26].

There is a long debate in the nanofluid community on the mechanisms of thermal conductivity of nanofluids. A number of theories have been proposed including the interfacial layering, Brownian motion, ballistic transport of energy carriers, the interfacial resistance, the structure effect, and particle aggregation and percolation effects [4]. As reviewed recently, the Brownian motion and its associated micro-convection as well as the interfacial layer mechanism would not be responsible. The effect of particle morphology in the liquid, i.e., through aggregation or percolation, has been proposed by a number of researchers recently [27,28]. Those studies emphasized that the enhancement of thermal conductivity was a function of nanoparticle aggregation and showed that there would exist an optimized aggregation structure to achieve the maximum thermal conductivity, which could be far beyond the prediction from homogeneous dispersions. A recent study reported a switchable thermal conductivity of ferrofluids through an external magnetic field by engineering particle morphology in the liquid [29]. An extraordinary enhancement of thermal conductivity, approximately 300%, is observed when linear chain-like percolating structures are generated and uniformly dispersed in the base fluid, while negligible enhancement is obtained for the well-dispersed particles. Similarly, Horton et al. [30] showed a time-dependent thermal conductivity of nickel-coated carbon nanotubes with the application of an external magnetic field, which was related to the structuring, percolation, and agglomeration of carbon nanotubes influenced by the magnetic field. At its peak value, the thermal conductivity was found to be approximately 80% higher than that of water. The difference in the absolute values in the enhancement might be related to different contributions from microstructures and the interfacial resistance. A recent simulation showed that the radius of gyration and particle-fluid interfacial area are the two important parameters in characterizing microstructures [31]. The increase in thermal conductivity due to the increase of the shape factor could be offset by a negative contribution of increased heat flow resistance at the solid-liquid interface [27]. Although further study is still needed, these studies illustrate that the effective thermal conductivity of nanofluids could be adjusted by proper control of the external magnetic field to generate different nanoparticle-percolating structures. Properly engineered, such an approach could open a new window for engineering unique nanofluid properties for different applications. It appears reasonable to conclude that different shaped nanoplates, as shown in Figure 4, might be responsible for the large thermal conductivity increase, although this still requires further detailed examination.

Although loose percolation structures may exclude thermal conductivity as an inherent physical property as they can be destroyed under flow and heating conditions, these studies did show that the particle morphology in the liquid could significantly affect the effective thermal conductivity. The potential influence of particle structure on thermal conduction emphasizes that colloid chemistry will play a significant role in optimizing the thermal conductivity of nanofluids. For instance, through the current nanoplate approach, the morphology will not be destroyed under any shearing or heating conditions. However, further careful examinations are still required regarding to the influence of particle morphology structure on other effective properties, especially surface tension, wettability, viscosity, and specific heat [32]. The gain from thermal conductivity could be offset by an increase in viscosity or interfacial resistance, and a decrease in specific heat. Other areas of future research should pay more attention to the linkage between the rheology and thermal properties, nanoparticle interactions and particle-fluid-surface interactions, which calls for interdisciplinary collaboration among nanomaterials, colloid science, and engineering researchers.

## Conclusions

Different gold nanofluids were produced from the one-step approach based on the Citrate Reduction (CR) method with the aid of ultrasonication for particle morphology control. The physical and thermal property characterizations show that

(1) different nanofluids containing different sizes and shapes of GNP can be engineered by properly controlling the reaction process.

(2) The ultrasonication is a very powerful tool in engineering particle size and shape. By applying ultrasonication, the spherical particle size can be controlled in the range of 10-20 nm, and the average size of gold nanoplate can be reduced from 300 to 50 nm, with more uniform and regular shapes.

(3) Large increase in the effective thermal conductivity of nanofluid is found for gold nanofluids, especially under relatively higher particle concentrations, i.e., >33 μM/L.

(4) The effective thermal conductivity of gold nanofluids is not only dependent on particle size, but also heavily influenced by particle shape, whereas further mechanistic understanding is required.

## Abbreviations

CR: citrate reduction; DLS: dynamic light scattering; EDX: energy dispersive X-ray spectroscope; GNP: gold nanoparticle; INPBE: International Nanofluid Properties Benchmark Exercise; PPT: plasmonic photothermal therapy; SEM: scanning electron microscope; SPR: surface plasmon resonance; TEM: transmission electron microscopy.

## Competing interests

The authors declare that they have no competing interests.

## Authors' contributions

HC performed experiments and helped to draft the manuscript. DW proposed idea, designed experiments and finalized the manuscript. All authors read and approved the manuscript.
